# Effect of stroma on the behavior of temoporfin-loaded lipid nanovesicles inside the stroma-rich head and neck carcinoma spheroids

**DOI:** 10.1186/s12951-020-00743-x

**Published:** 2021-01-06

**Authors:** Ilya Yakavets, Aurelie Francois, Laureline Lamy, Max Piffoux, Florence Gazeau, Claire Wilhelm, Vladimir Zorin, Amanda K. A. Silva, Lina Bezdetnaya

**Affiliations:** 1 Centre de Recherche en Automatique de Nancy, Centre National de la Recherche Scientifique, UMR 7039, Université de Lorraine, Campus Sciences, Boulevard des Aiguillette, 54506 Vandoeuvre-lès-Nancy, France; 2 Present Address: Research Department, Institut de Cancérologie de Lorraine, 6 avenue de Bourgogne, 54519 Vandoeuvre-lès-Nancy, France; 3 Laboratoire Matière et systèmes complexes, CNRS UMR 7057, Université de Paris, 75205 Paris Cedex 13, France; 4 Laboratory of Biophysics and Biotechnology, Belarusian State University, 4 Nezavisimosti Avenue, 220030 Minsk, Belarus

**Keywords:** Temoporfin, Extracellular vesicles, Drug-in-cyclodextrin-in-liposomes, Multicellular tumor spheroids, Extracellular matrix, Drug penetration, Photodynamic therapy

## Abstract

**Background:**

Despite the highly expected clinical application of nanoparticles (NPs), the translation of NPs from lab to the clinic has been relatively slow. Co-culture 3D spheroids account for the 3D arrangement of tumor cells and stromal components, e.g., cancer-associated fibroblasts (CAFs) and extracellular matrix, recapitulating microenvironment of head and neck squamous cell carcinoma (HNSCC). In the present study, we investigated how the stroma-rich tumor microenvironment affects the uptake, penetration, and photodynamic efficiency of three lipid-based nanoformulations of approved in EU photosensitizer temoporfin (mTHPC): Foslip^®^ (mTHPC in conventional liposomes), drug-in-cyclodextrin-in-liposomes (mTHPC-DCL) and extracellular vesicles (mTHPC-EVs).

**Results:**

Collagen expression in co-culture stroma-rich 3D HNSCC spheroids correlates with the amount of CAFs (MeWo cells) in individual spheroid. The assessment of mTHPC loading demonstrated that Foslip^®^, mTHPC-DCL and mTHPC-EVs encapsulated 0.05 × 10^− 15^ g, 0.07 × 10^− 15^ g, and 1.3 × 10^− 15^ g of mTHPC per nanovesicle, respectively. The mid-penetration depth of mTHPC NPs in spheroids was 47.8 µm (Foslip^®^), 87.8 µm (mTHPC-DCL), and 49.7 µm (mTHPC-EVs), irrespective of the percentage of stromal components. The cellular uptake of Foslip^®^ and mTHPC-DCL was significantly higher in stroma-rich co-culture spheroids and was increasing upon the addition of serum in the culture medium. Importantly, we observed no significant difference between PDT effect in monoculture and co-culture spheroids treated with lipid-based NPs. Overall, in all types of spheroids mTHPC-EVs demonstrated outstanding total cellular uptake and PDT efficiency comparable to other NPs.

**Conclusions:**

The stromal microenvironment strongly affects the uptake of NPs, while the penetration and PDT efficacy are less sensitive to the presence of stromal components. mTHPC-EVs outperform other lipid nanovesicles due to the extremely high loading capacity. The results of the present study enlarge our understanding of how stroma components affect the delivery of NPs into the tumors. 
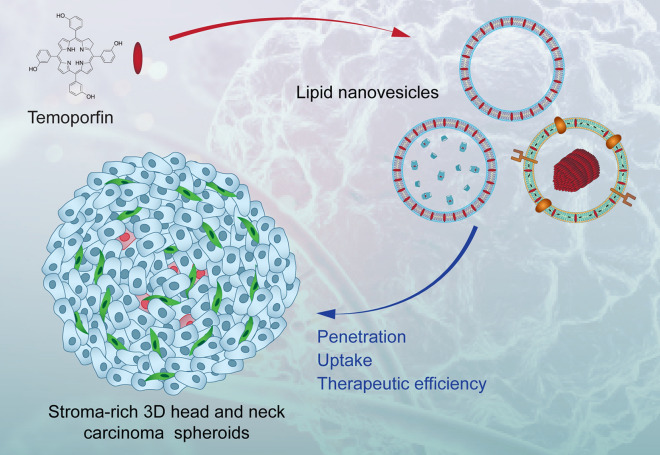

## Background

For many years, nanomedicine has evolved as a key technology in the delivery of cancer drugs [1]. Nanoparticles (NPs) offer the possibility to improve pharmacokinetic parameters, resulting in better drug distribution, increased circulation time, targeted controlled release, increased intracellular concentration, and enhanced solubility and stability of drugs in the organism [2, 3]. Much of the justification for the use of NPs is based on the enhanced permeability and retention effect, which proposes NPs preferable accumulation in tumors due to a leaky vasculature and suppresses lymphatic drainage [4]. Commonly, cellular interactions of the developed nanomaterials are assessed in 2D culture monolayers *in vitro*, which lack critical stroma components of the tumor microenvironment (TME) [5]. Thus, there is a need to develop a better understanding of how stroma components affect the delivery of NPs into the tumors.

3D *in vitro* tumor models (i.e., multilayers, spheroids, microtissues) recapitulate several aspects of TME, providing better predictive results, and facilitating clinical translation [6]. Most of 3D *in vitro* tumor models are focusing only on tumor cells, missing stroma components, e.g., extracellular matrix (ECM) [6], which are considered as a physical barrier for NPs penetration into the tumors [5, 7]. Recently, we reported the optimization of sophisticated 3D co-culture tumor spheroids consisting of head and neck squamous carcinoma cells (HNSCC) and cancer-associated fibroblasts (CAFs), capable of recapitulating various levels of ECM expression in the HNSCC [8]. Using this model, we have demonstrated that the presence of stroma content in 3D HNSCC spheroids influences the behavior of photoactive drugs in different ways.

Temoporfin (5,10,15,20-Tetrakis(3-hydroxyphenyl)chlorin, mTHPC) is a highly efficient photosensitizer (PS), approved for palliative photodynamic therapy (PDT) of advanced HNSCC [9]. In our recent review [10], we reported that lipid-based nanodelivery systems are superior to other types of NPs in the delivery of mTHPC. For instance, mTHPC liposomal formulation Foslip®, the most studied mTHPC nanoformulation, demonstrated improved bioavailability [11], better tumor selectivity, and shorter drug-light interval achieving efficient PDT [12, 13]. A novel perspective biogenic delivery nanoplatforms based on naturally-derived extracellular vesicles (mTHPC-EVs) have been recently investigated, showing improved plasma stability and increased PDT efficiency compared with Foslip^®^ [14]. Meanwhile, drug-in-cyclodextrin-in-liposomes (mTHPC-DCL) have been recently proposed to ensure the deep penetration of mTHPC into the tumor tissue [15, 16].

The objective of the present study was to investigate the effect of stroma-rich tumor microenvironment on the uptake, penetration, and photodynamic efficiency of three mTHPC-loaded nanovesicles (e.g., Foslip^®^, mTHPC-DCL, and mTHPC-EVs). Stroma-rich tumor microenvironment was recapitulated using co-culture HNSCC spheroids consisting of FaDu (human pharynx squamous cell carcinoma) and MeWo (CAF, granular fibroblasts, derived from human melanoma) [17]. We characterized the expression of ECM macromolecules in spheroids as a function of CAF concentration and demonstrated its correlation with uptake and penetration of mTHPC nanovesicles.

## Results and discussion

### Characterization of co-culture HNSCC model

**Fig. 1:**
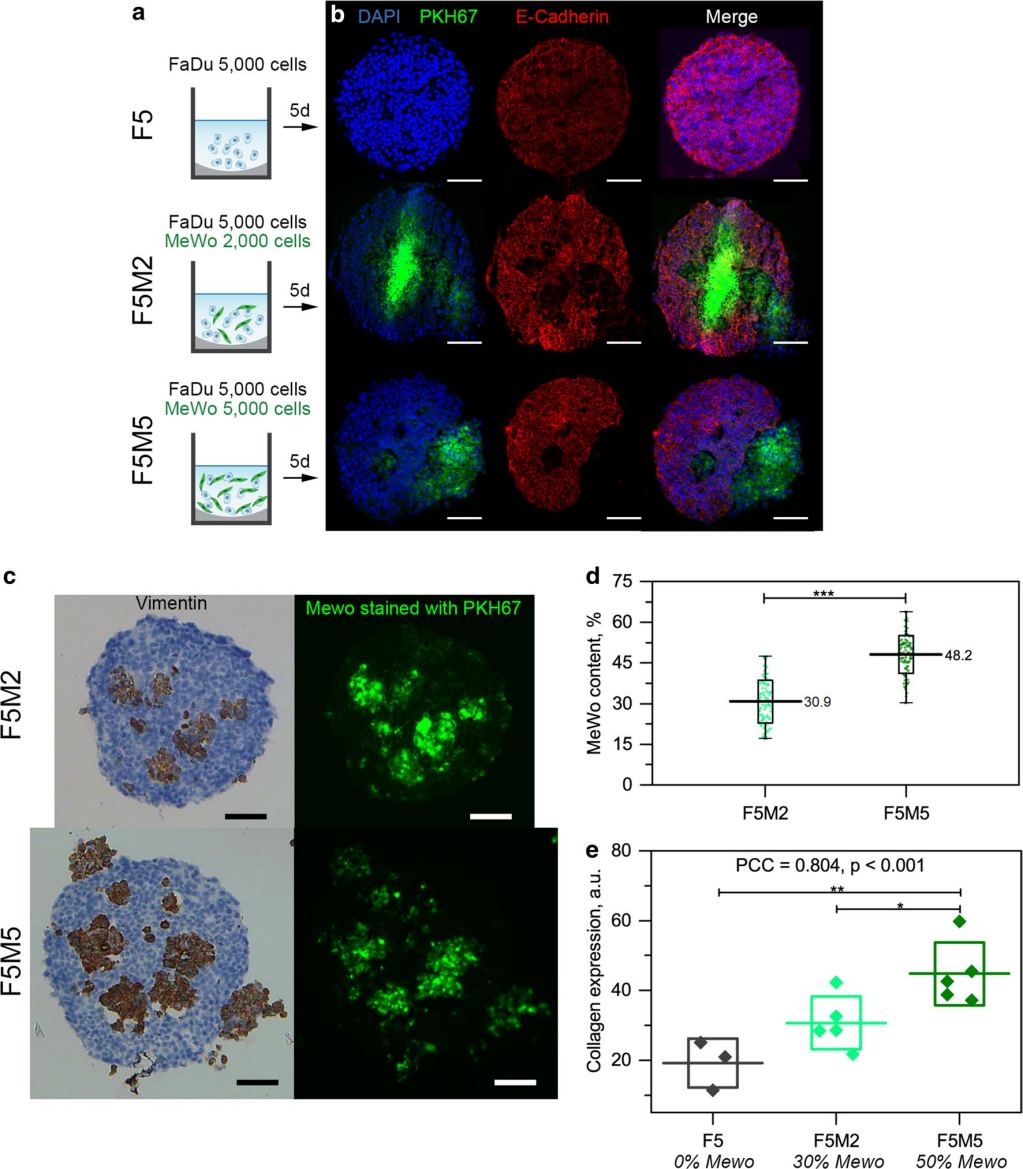
Characterization of 3D HNSCC spheroids.** a** Schematic representation of cell seeding used for the formation of F5, F5M2 and F5M5 spheroids. **b, c** Typical microscopy images of cryosections of homo- (F5) and heterospheroids (F5M2 and F5M5) at day 5 post-seeding, stained with (**b**) DAPI nuclear dye, PKH67 and E-Cadherin markers and (**c**) vimentin and collagen markers. MeWo cells were pre-stained with PKH67 membrane dye. **d** Colocalization of vimentin and MeWo cells, stained with PKH67, within co-culture spheroids. Scale bar – 100 µm. **e** The percentage of MeWo cells in F5M2 ( ) and F5M5 ( ) co-culture spheroids 5 days post-seeding. The graph represents the mean ± SD (box) together with minimum and maximum limits (whiskers) [n = 87; ***p < 0.001, using two-sample t-test]. **f** Semi-quantitative image analysis of collagen expression in F5 ( ), F5M2 ( ), and F5M5 ( ) spheroids. Data presented as mean ± SD [n = 3–5; *p < 0.05; **p < 0.01, using ANOVA]

Co-culture spheroids from FaDu (tumor cells) and MeWo (CAF) cells were generated in order to address the interaction of NPs with HNSCC stroma (Fig. 1a). Using the liquid overlay technique [8], we formed monoculture F5 spheroids (5000 FaDu cells per well), and co-culture spheroids, denoted as F5M2 (5000 FaDu + 2000 MeWo cells) and F5M5 (5000 FaDu + 5000 MeWo cells). After 5 days, FaDu spheroids had a uniform spherical shape with a high cell density most probably due to the strong expression of E-Cadherin adhesion protein (Fig. 1b). In co-culture spheroids, MeWo cells were stained with PKH67 green membrane dye before seeding with FaDu cells in order to distinguish tumor cells and CAFs. MeWo cells formed large clusters within spheroids and did not express E-Cadherin (red color, Fig. 1b), making spheroids more fragile. Moreover, MeWo cells (green color) strongly express vimentin (brown color, Fig. 1c). Using flow cytometry analysis of cell suspension from the dissociated spheroids, we estimated that the fraction of MeWo cells was 30.9 ± 7.9% and 48.2 ± 7.0% in F5M2 and F5M5 co-culture spheroids, respectively (Fig. 1d). Based on our previous work, where we presented the images of spheroids stained with Picro Sirius Red (collagen staining) [8], we performed a semi-quantitative analysis of collagen expression (Fig. 1e). Given the calculated fraction of MeWo cells, we confirmed a strong correlation between MeWo content and ECM expression (Pearson correlation coefficient (PCC) = 0.8; *p* < 0.001) in generated spheroids.

### Lipid-based mTHPC nanovesicles

**Fig. 2:**
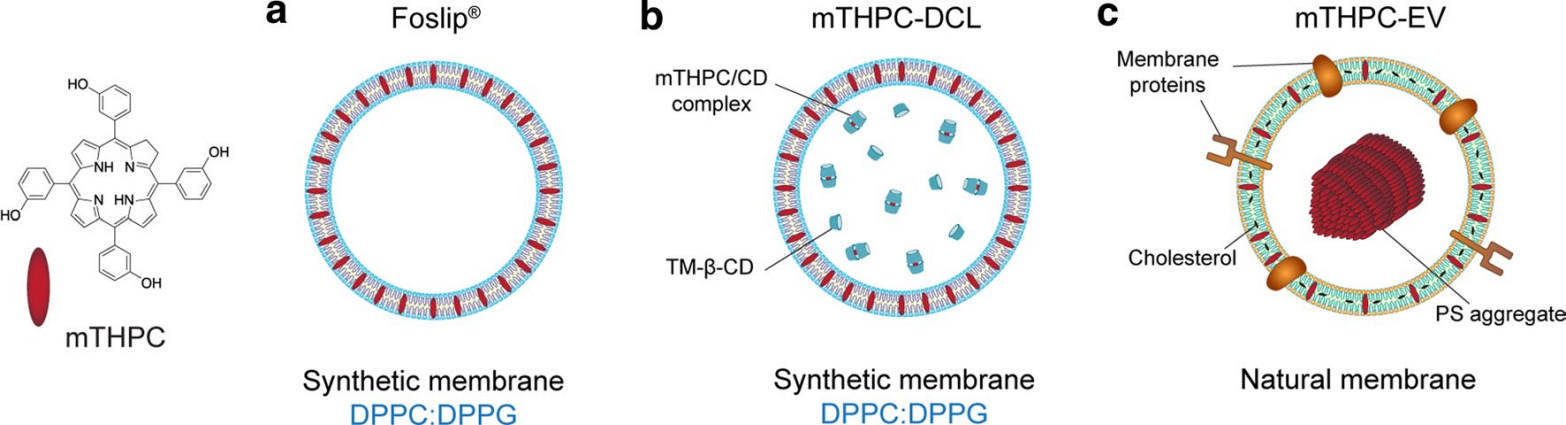
Schematic representations of lipid-based mTHPC nanoformulation: **a** Foslip^®^, **b** mTHPC-DCL, **c** mTHPC-EV

**Fig. 3:**
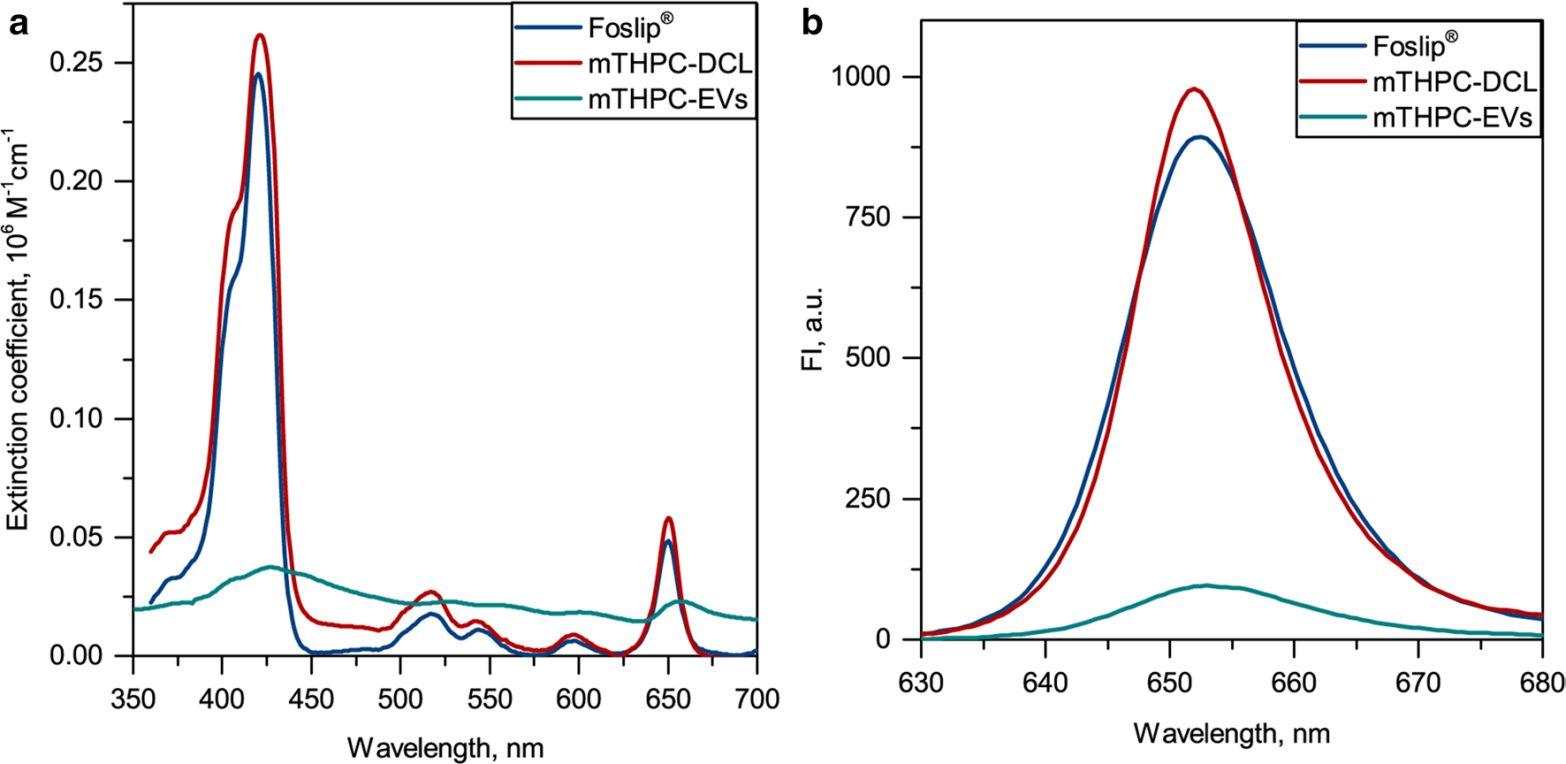
Absorbance and fluorescence emission spectra of mTHPC in nanovesicles. The mTHPC concentration was 1 µM. The fluorescence was excited at 420 nm

In this study, we tested conventional mTHPC liposomes (Foslip^®^), mTHPC-in-cyclodextrin-in-liposomes (mTHPC-DCLs) and mTHPC-loaded naturally-derived extracellular vesicles (mTHPC-EVs) (Fig. 2). As seen from the absorption and fluorescence spectra (Fig. 3), Foslip^®^ and mTHPC-DCLs exhibit narrow spectral bands corresponding to the monomeric PS in both lipid bilayer and cyclodextrin complexes. Meanwhile, the absorbance and fluorescence emission spectra of mTHPC-EVs were dramatically decreased, most probably indicating that a substantial part of mTHPC molecules is in an aggregated state in the aqueous lumen of EVs. We have estimated that the fluorescence quantum yield of mTHPC-EVs was twice lower than these of Foslip^®^ or mTHPC-DCLs. This partial aggregation state of mTHPC-EVs was confirmed in our recent paper by measuring the photoinduced fluorescence quenching [18]. Compared to Foslip^®^, where mTHPC is directly loaded in lipid suspension, mTHPC-EVs were produced by turbulence-triggered technique, which implies the incubation of HUVEC cells in bioreactors in the serum-free DMEM medium containing 100 µM of free mTHPC. Thus, it is highly probable that during the production, EVs captured mTHPC aggregates, which stay inside EVs even after purification and isolation procedures. Given that, we could speculate that mTHPC loading capacity in mTHPC-EVs is much higher than that of Foslip®. In fact, according to the NP tracking analysis, there are 5.5 mg/ml of mTHPC in 4.2 × 10^12^ particles/ml for mTHPC-EVs [18], while in the case of Foslip^®^, 3.2 × 10^12^ particles/ml encapsulated 0.15 mg/ml of mTHPC (data not shown). According to a previous report, the loading of mTHPC-DCL is 150% compared to Foslip^®^ [16]. Given that, we deduced that Foslip^®^, mTHPC-DCL, and mTHPC-EV encapsulated 0.05 × 10^− 15^ g, 0.07 × 10^− 15^ g, and 1.3 × 10^− 15^ g of mTHPC per NP, respectively.

### Penetration into spheroids

**Fig. 4:**
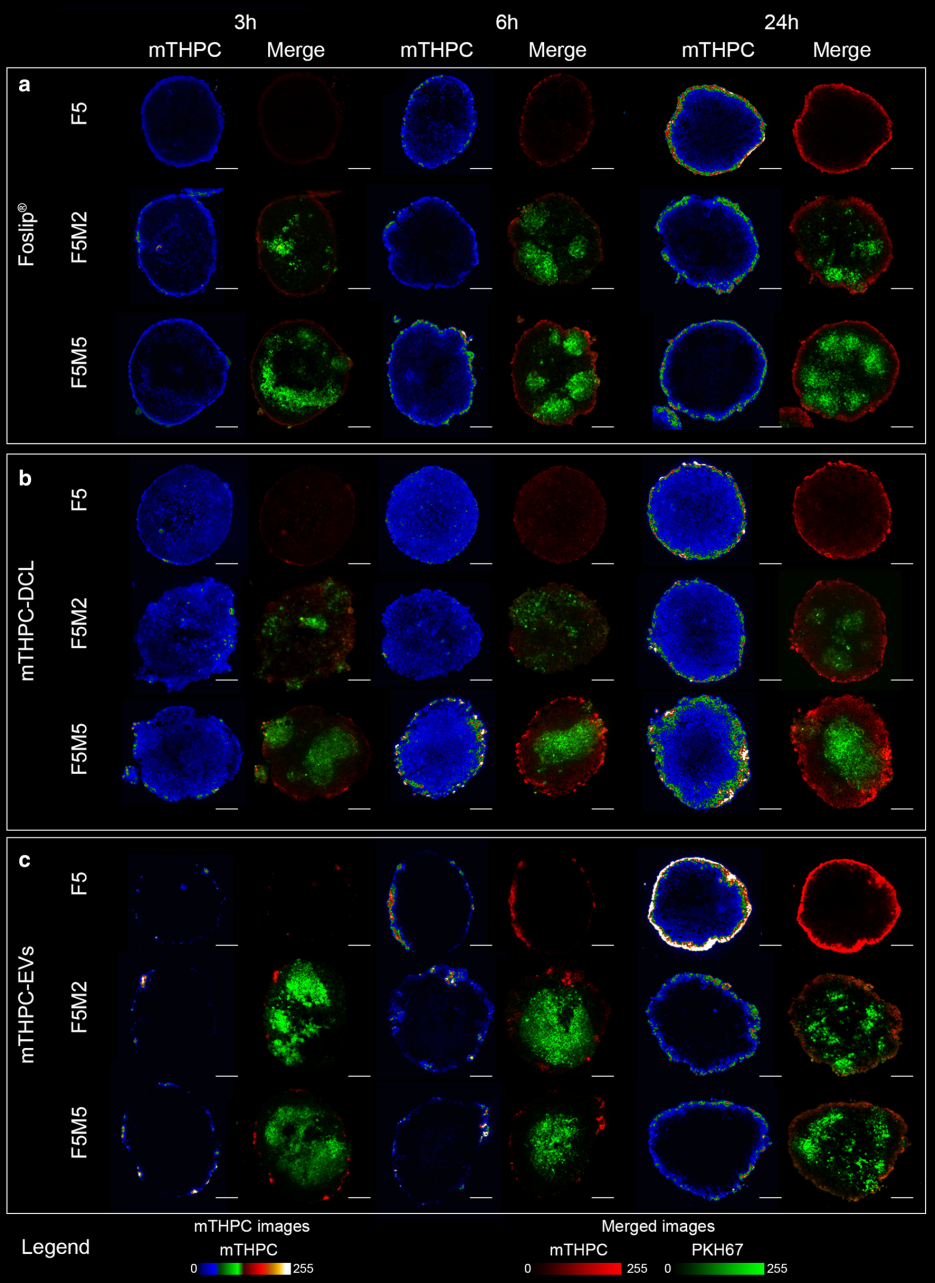
Penetration of mTHPC-loaded NPs in co-culture spheroids. The typical fluorescence images of cryosections of FaDu monoculture (F5) and FaDu:MeWo (5:2 and 5:5) co-culture spheroids at day 5 post-seeding after incubation with (**a**) Foslip^®^, (**b**) mTHPC-DCL and (**c**) mTHPC-EVs for 3, 6 and 24 h. MeWo cells were pre-stained with PKH67 membrane dye (green color). mTHPC fluorescence is displayed in red color (merged images) and pseudo-colors (mTHPC images). The concentration of mTHPC was 4.5 µM. Serum concentration – 6%. Scale bar – 100 µm

**Fig. 5:**
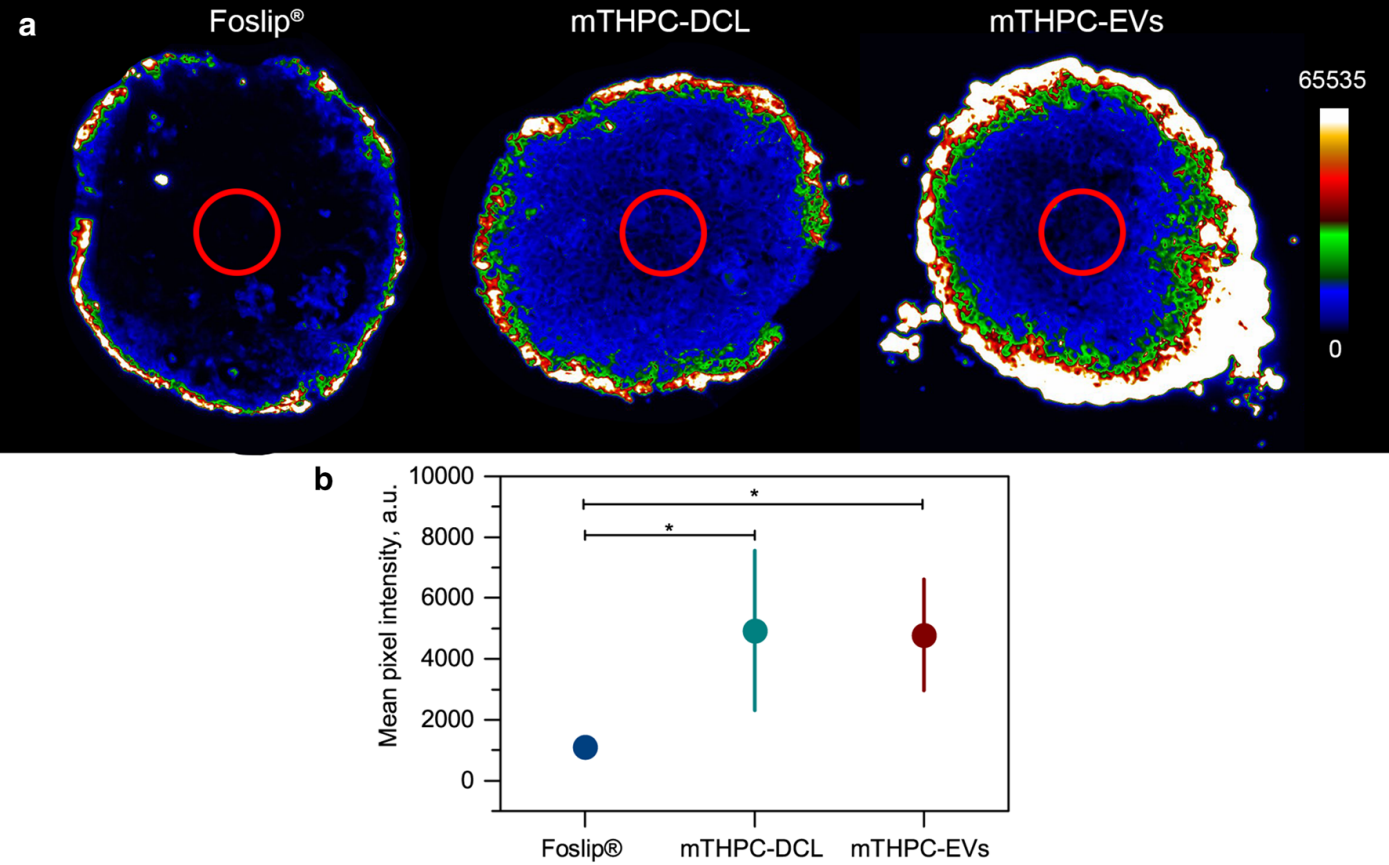
Head-to-head comparison of penetration of mTHPC-loaded nanovesicles in F5M5 spheroids.** a** The typical fluorescence images of cryosections of co-culture (F5M5) spheroids at day 5 post-seeding after incubation with Foslip^®^, mTHPC-DCL and mTHPC-EVs for 24 h. mTHPC fluorescence was measured at equal exposure time for all NPs and is displayed in pseudo-colors. The concentration of mTHPC was 4.5 µM. Serum concentration – 6%. Scale bar – 100 µm. **b** The quantification analysis of mTHPC fluorescence intensity in F5M5 spheroid’ core (red circles) after 24 h incubation with Foslip^®^, mTHPC-DCL and mTHPC-EVs. Data presented as mean ± SD [n = 4–7; *p < 0.05; *p < 0.01 and *p < 0.001, using ANOVA]

Fluorescence microscopy of cryosections was used to study the penetration of mTHPC-loaded nanovesicles in stroma-rich co-culture spheroids. Figure 4 displays the typical fluorescence images of spheroid’s cryosections after 3 h, 6 h, and 24 h incubation with Foslip^®^ (Panel A), mTHPC-DCLs (Panel B), and mTHPC-EVs (Panel C). The fluorescence of Foslip^®^ was limited to the external rim of spheroids (Fig. 4a). Over time, the fluorescence intensity in peripheral cells increases, without appreciable PS penetration in the inner core of spheroids. It is worth noting that superficial localization of Foslip^®^ has been already reported for several types of 3D cell cultures [14, 16, 18–20], and thus was anticipated in FaDu spheroids. Peripheral fluorescence pattern of Foslip^®^ also persisted in FaDu/MeWo spheroids, which are rich in ECM components. mTHPC is tightly sequestered in cells, significantly decreasing the probability of intracellular transport of mTHPC within spheroids and, as such, resulting in heterogeneous accumulation of mTHPC only in the outermost peripherical cell layers [8, 14, 19]. In contrast, mTHPC-DCL easily penetrates into FaDu and FaDu/MeWo spheroids from 3 h incubation, demonstrating higher mTHPC fluorescence in the core of spheroids (Fig. 4b). As pre-staining of MeWo cells allows assessing the selectivity of NPs uptake in merged images, no obvious selectivity of mTHPC-DCL toward MeWo cells was observed. The total mTHPC concentration delivered by mTHPC-EVs to spheroids was much higher than these of Foslip^®^ and mTHPC-DCLs, resulting in the saturation of images. Obviously, saturated images could not allow visualization of the data required for a quantitative comparison of penetration profiles. Thus, we adjusted the acquisition settings for cryosections exposed to mTHPC-EVs, avoiding the saturation of the pixels (Fig. 4c). In fact, similarly to Foslip^®^, mTHPC-EVs accumulated in the outer rim of spheroids. At 3 h and 6 h incubation, a spotted fluorescent signal of mTHPC was observed on the surface of spheroids, while at 24 h continuous mTHPC fluorescence was mostly localized in the external cell rim of spheroids. In order to perform a head-to-head comparison of NPs accumulation in the central zone of F5M5 spheroids, we compared images collected in the equal acquisition settings (Fig. 5a). We quantified and compared mTHPC fluorescence intensity in the central zone (red circle) of spheroids treated with different NPs. We demonstrated that mTHPC-EVs and mTHPC-DCLs deliver a significantly higher amount of mTHPC to the center of spheroids than Foslip^®^ (*p* < 0.05) (Fig. 5b). These data are consistent with the recent report, where the authors demonstrated the concentration-depended penetration of doxorubicin in spheroids [21]. Visually, there was no difference in penetration depth in stroma-rich spheroids, despite the general consideration of ECM as a physical barrier for NPs [5, 22, 23]. However, it is rather difficult to distinguish the differences between mono-and co-culture spheroids without the quantification analysis.

**Fig. 6:**
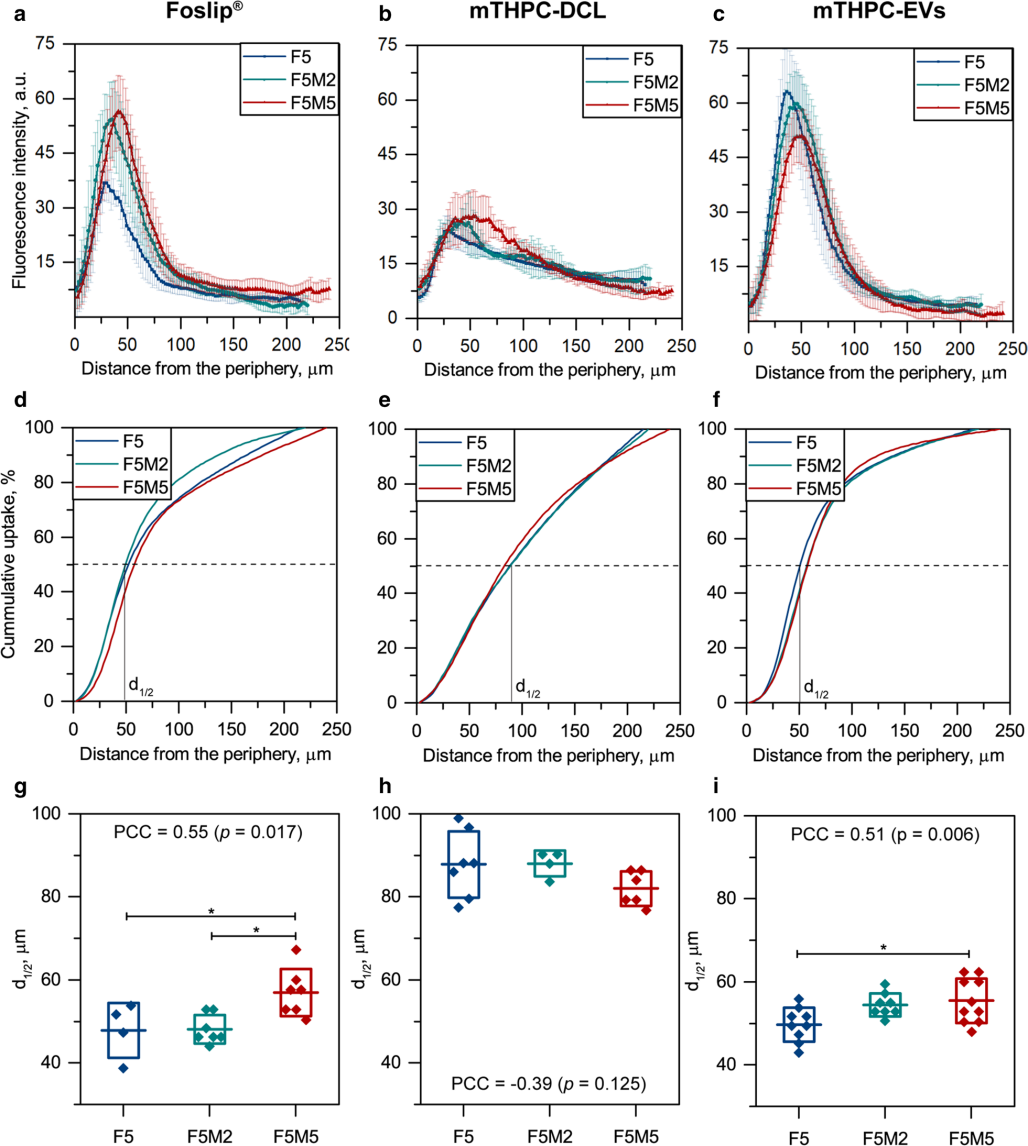
The quantification analysis of NPs penetration in spheroids at 24 h incubation. **a–c** The fluorescence profiles, as a function of distance from the spheroid periphery. Fluorescence intensity is presented as mean ± SD. **d–f** The cumulative uptake curves of mTHPC-loaded NPs in F5 (blue), F5M2 (green), and F5M5 (red) spheroids. The curve is presented as a mean of 4–10 profiles. **g–i** The mid penetration depth (d_1/2_) of NPs in F5 ( ), F5M2 ( ), and F5M5 ( ) spheroids. Data presented as mean ± SD (box) [n = 4–10; *p < 0.05, using ANOVA]

Therefore, we further conducted the quantification analysis of fluorescence imaging data for 24 h incubation time point (Fig. 6). The fluorescence profiles of NPs, as a function of distance from the spheroid periphery, were obtained using a custom script in ImageJ (Fig. 6a–c). In the cases of Foslip^®^ (Fig. 6a) and mTHPC-EVs (Fig. 6c), the penetration profiles demonstrated that mTHPC fluorescence signal dramatically decreases from the periphery towards the center of the spheroids. mTHPC-DCL has a much smoother profile (Fig. 6b), confirming deep penetration into spheroids. In addition, it seems that the fluorescence intensity of Foslip^®^ and mTHPC-DCLs was higher in co-culture spheroids, while the signal of mTHPC-EVs was lower in F5M5 spheroids compared to F5 ones. To quantify the relationships between NPs penetration and stroma content in FaDu/MeWo spheroids, we calculated the cumulative accumulation curves (Fig. 6d–f), and estimated the depth along the radius in the spheroid, where PS concentration decreased by half, denoting it as mid-penetration depth (d_1/2_) of NPs (Fig. 6g–i). Indeed, the cumulative uptake curves for Foslip^®^ and mTHPC-EVs (Fig. 6d, f) were increasing rapidly, reaching 50% of mTHPC uptake between 40 and 60 µm for all types of spheroids. Meanwhile, the cumulative uptake curve of mTHPC-DCL was close to linear, demonstrating a quite uniform distribution of mTHPC across the spheroid independently on the type of spheroids (Fig. 6e). In fact, d_1/2_ of mTHPC-DCLs in monoculture F5 spheroids was 87.8 ± 8.0 µm (Fig. 6h), being significantly higher (*p* < 0.001) than that of Foslip^®^ and mTHPC-EVs (47.8 ± 6.7 µm and 49.7 ± 4.1 µm). On the other hand, the d_1/2_ of Foslip^®^ and mTHPC-EVs was significantly higher in F5M5 spheroids compared to F5 spheroids (Fig. 6g, i). The calculated PCCs between the mid-penetration depth (d_1/2_) and concentration of MeWo cells in spheroids were 0.51 and 0.55 (*p* < 0.05) for Foslip^®^ and mTHPC-EVs, respectively. Meanwhile, for mTHPC-DCLs, the correlation between d_1/2_ and stroma content was not statistically significant (PCC = − 0.39; *p* = 0.125) (Fig. 6h). Important to note that penetration of NPs normalized to spheroids’ size (0–100%) was independent on the type of spheroids (data not shown).

It was previously reported that mTHPC-EVs penetrate deeper compared to Foslip^®^ in HT29 spheroids [18], while in FaDu spheroids, both nanovesicles have quite similar penetration ability. We hypothesized that FaDu cells, which strongly express E-Cadherin (Fig. 1), are more tightly packed in spheroids than HT29 cells, complicating the penetration of NPs to the spheroid’s depth. In turn, MeWo cells did not express E-Cadherin (cell junction protein) (Fig. 1), making co-culture spheroids more gaping, while the dense collagen matrix acts as a physical barrier, compensating this loose effect and resulting in a similar penetration of NPs. In the case of free PS, the barrier effect is not that important, explaining the fact of increased penetration of free mTHPC in stroma-rich spheroids [8]. Concerning the mTHPC NPs, to date, only a limited number of NPs [10], preferably cyclodextrin-based [15, 16, 24, 25], demonstrated the deep penetration in 3D cancer models. There is increasing evidence indicating that the physicochemical properties of NPs, such as size, charge, and surface chemistry, play a crucial role in their ability to penetrate ECM [5, 6, 26]. NPs tested in this study possess close physicochemical characteristics, ca. 100–200 nm, negative charge up to – 38 mV [15, 18], allowing comparison of their behavior in 3D tumor spheroids. Based on theoretical models, the size and surface properties (e.g., charge and morphology) of NPs are considered to be crucial for penetration into solid tumors [5, 27–29]. For instance, small (< 60 nm) and neutral (± 10 mV) NPs are preferred for deep penetration into the dense positively charged collagen matrix [22, 23]. At the same time, the consideration of these parameters for designing lipid vesicles could result in reduced colloidal stability and lower encapsulation capacity of liposomes. Moreover, it is hardly possible to produce EVs with uniform physicochemical characteristics due to their natural origin. In the case of mTHPC-DCL, mTHPC/cyclodextrin nanoshuttles (ca. 2–3 nm) released from vesicles provide deep penetration of mTHPC-DCLs, as was confirmed for HT29 spheroids by chromatography technique [16]. Based on quantification analysis, such a mechanism is supposed to be independent on the ECM content in spheroids.

### Uptake of lipid nanovesicles in individual cells of spheroids

**Fig. 7:**
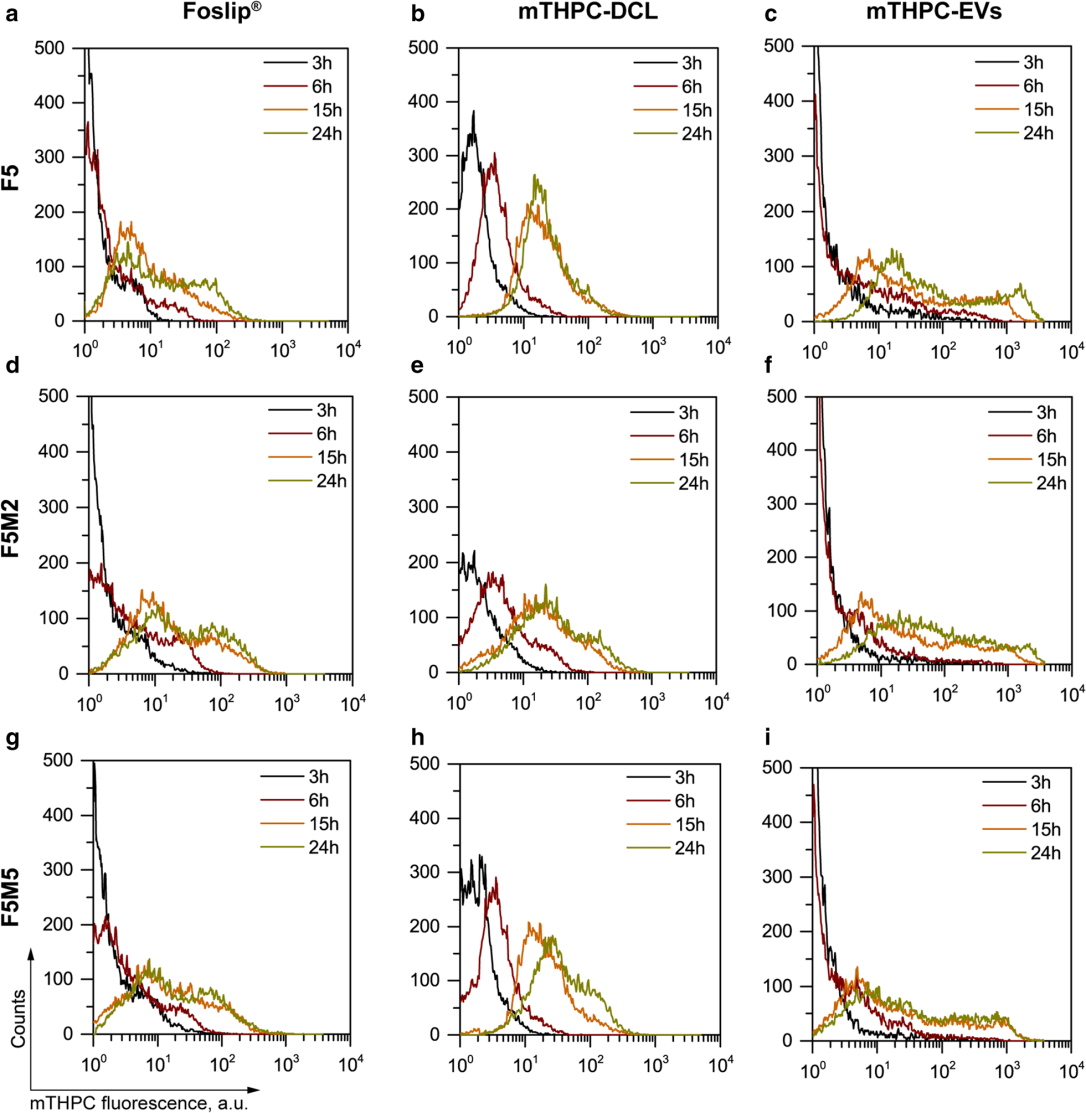
Kinetics of cellular accumulation of mTHPC-loaded NPs in co-culture spheroids. Typical flow cytometry histograms of dissociated (**a**, **b**, **c**) F5, (**d**, **e**, **f**) F5M2 and (**g**, **h**, **i**) F5M5 spheroids treated with (**a**, **d**, **g**) Foslip^®^, (**b**, **e**, **h**) TD and (**c**, **f**, **i**) mTHPC-EVs at 3 (black), 6 (red), 15 (orange) and 24 h (dark yellow) post-incubation

Fluorescence imaging suggested that the expression of stroma in co-culture spheroids possibly affects the total uptake of NPs. In order to accurately estimate the influence of stroma content on the accumulation of mTHPC-loaded nanovesicles in individual cells of spheroids, we analyzed cell suspensions after spheroids’ disintegration. The typical histograms from 20,000 cells of these suspensions were plotted for each time interval (Fig. 7a–i). In the case of F5 spheroids loaded with Foslip^®^ and mTHPC-EVs, the histograms possessed multiple peaks displaying the heterogeneity of PS distribution, while the distribution of mTHPC-DCL between individual cells in spheroids is more homogeneous (single peak histogram) (Fig. 7b). In stroma-rich spheroids, the second peak appeared in the mTHPC-DCL distribution (Fig. 7e, h). This effect is likely related to the preferential accumulation of mTHPC-DCLs in MeWo, as will be described further.

**Fig. 8:**
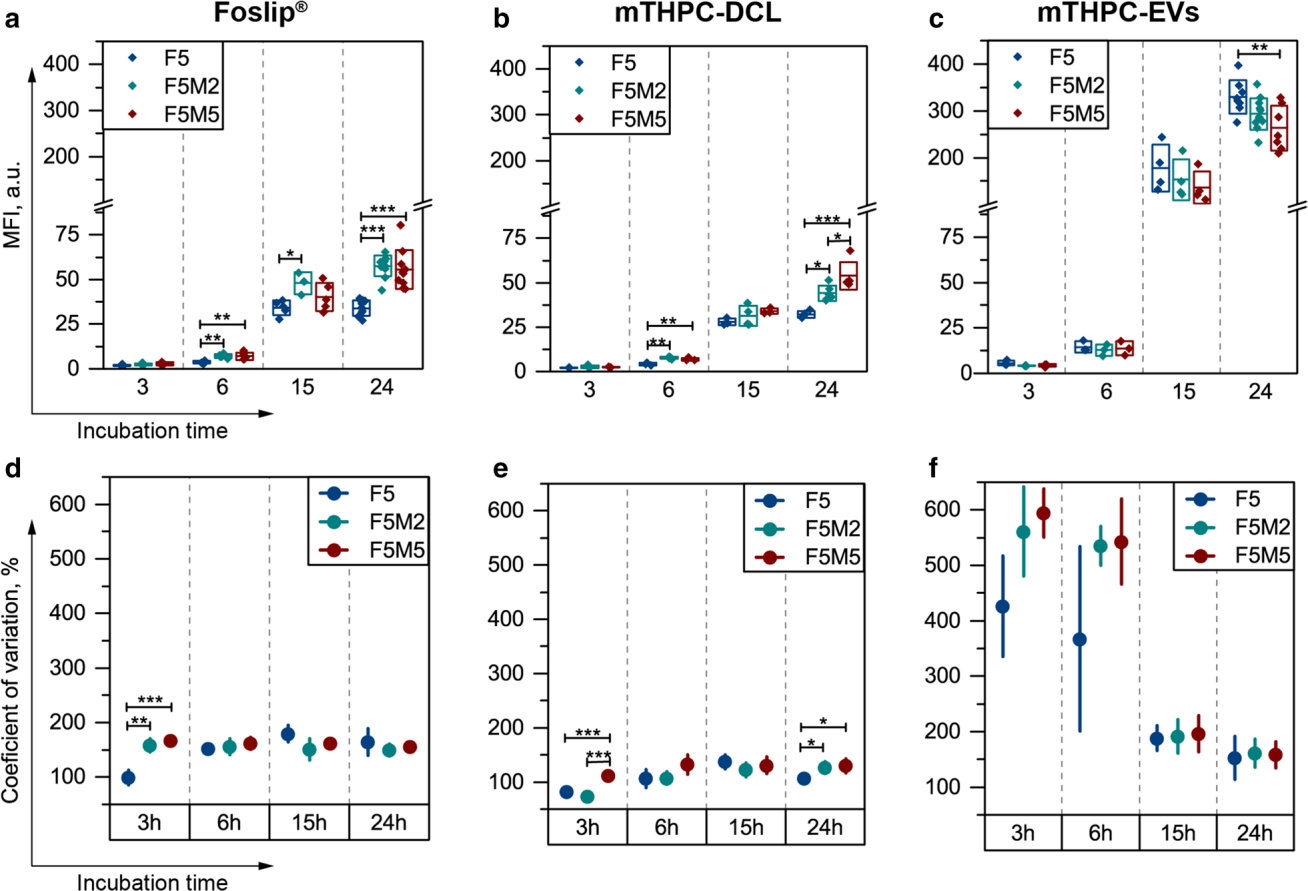
Cellular uptake of mTHPC-loaded nanovesicles in co-culture spheroids. Kinetics of mean fluorescence intensity (MFI) of mTHPC histograms in F5 ( ), F5M2 ( ) and F5M5 ( ) spheroid individual cells after 3 h, 6 h, 15 h and 24 h incubation with (**a**) Foslip^®^, (**b**) mTHPC-DCL and (**c**) mTHPC-EVs. **d–f** Coefficients of variation of flow cytometry histograms of spheroids in the function of time. The mTHPC concentration was 4.5 µM. Serum concentration – 6%. Data presented as mean ± SD [n = 3–10; *p < 0.05; *p < 0.01 and *p < 0.001, using ANOVA for each time point]

**Fig. 9:**
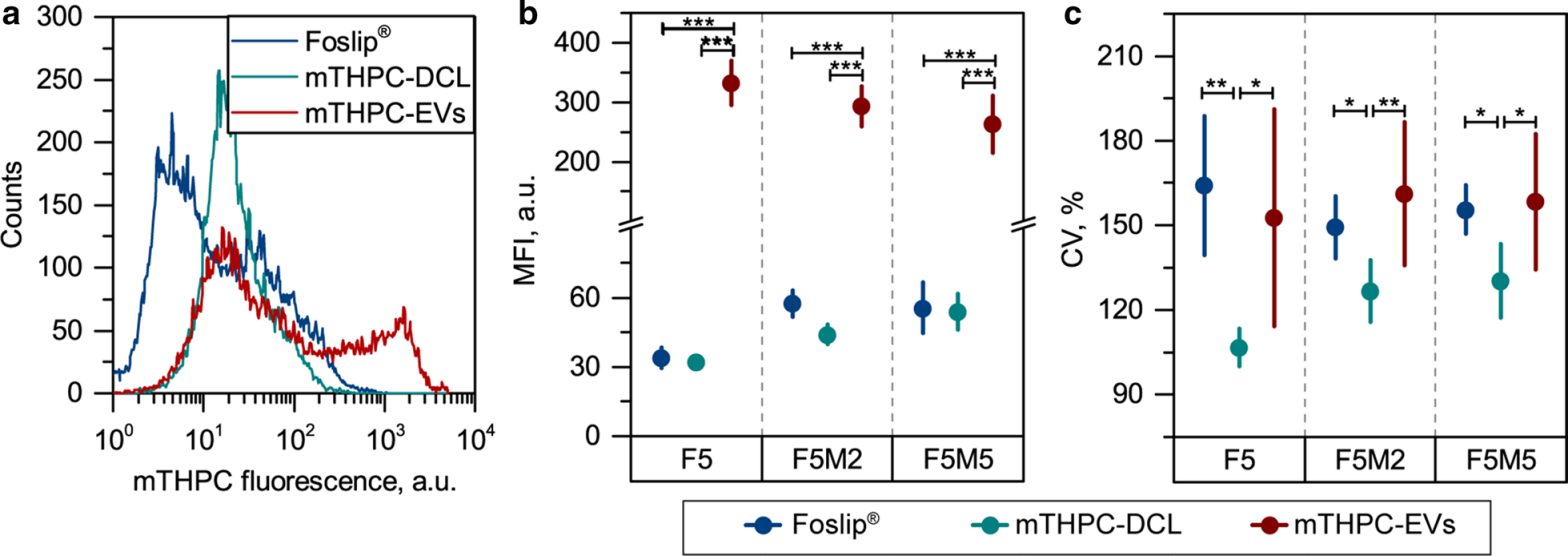
Head-to-head comparison of cellular uptake of mTHPC-loaded nanovesicles in spheroids. **a** Typical flow cytometry histograms of disassociated FaDu spheroids treated with Foslip^®^ (blue), mTHPC-DCL (cyan) and mTHPC-EVs (red) at 24 h post-incubation. **b** MFI and (**c**) coefficients of variation (CV) of flow cytometry histograms at 24 h of spheroids exposed to Foslip^®^ ( ), mTHPC-DCL ( ) and mTHPC-EVs ( ). The mTHPC concentration was 4.5 µM. Serum concentration – 6%. Data presented as mean ± SD [n = 5–10; *p < 0.05; *p < 0.01 and *p < 0.001, using ANOVA for each type of spheroids]

The cumulative uptake kinetics are expressed as mean fluorescence intensity (MFI) (Fig. 8a–c), while the coefficient of variation (CV) was used to assess the width of PS distribution (Fig. 8d–f). The uptake of Foslip® gradually increased from 6 h in all types of spheroids. Importantly, we observed significantly higher MFI in stroma-rich co-culture spheroids (F5M2 and F5M5) compared to the monoculture model (Fig. 8a). Concerning the shape of the histograms, they were quite wide with CV more than 150% starting from 6 h incubation (Fig. 8d). It worth noting that the CV of histograms was independent on the spheroid type. Meanwhile, mTHPC-DCL is also better accumulates in co-culture spheroids, as was shown at 6 h and 24 h time intervals (Fig. 8b). The distribution of mTHPC-DCL between individual cells of F5 spheroids was quite narrow (CV = 106%) (Fig. 8e). Concerning the effect of stroma, the secondary peak was observed in the histograms of co-culture spheroids treated with mTHPC-DCL (Fig. 7h), resulting in increased cellular uptake and higher CV of distribution (Fig. 8e). At last, we observed intensive accumulation of mTHPC-EVs in all types of spheroids after 15 h of incubation (Fig. 8c). mTHPC-EVs distribution was very heterogeneous in the first 6 h (CV > 400%), while the CV was about 200% and 150% for all types of spheroids at 15 and 24 h, respectively (Fig. 8f). Contrary to Foslip^®^ and mTHPC-DCL, where stroma positively influences cellular uptake, cumulative uptake of mTHPC-EVs was significantly lower at 24 h in F5M5 than that in monoculture F5 spheroids. The comparison of mTHPC NPs is presented in Fig. 9. Overall, mTHPC-DCL displayed the most homogeneous distribution (Fig. 9a) with the lowest CV (*p* < 0.05) (Fig. 9c), while the highest mTHPC accumulation was provided by naturally-derived mTHPC-EVs (almost ten times higher MFI, *p* < 0.001) (Fig. 9b). In fact, mTHPC-EVs should simultaneously transport an enormous amount of mTHPC, hidden as non-fluorescent PS aggregates in the aqueous lumen of EVs. The dissolution of PS aggregates in cells takes several hours, resulting in an increase in cellular fluorescence after 15 h of incubation with mTHPC-EVs. It worth noting that the cumulative cellular uptake of mTHPC-DCL was also equal to that of Foslip^®^ in all types of spheroids (Fig. 9b).

**Fig. 10:**
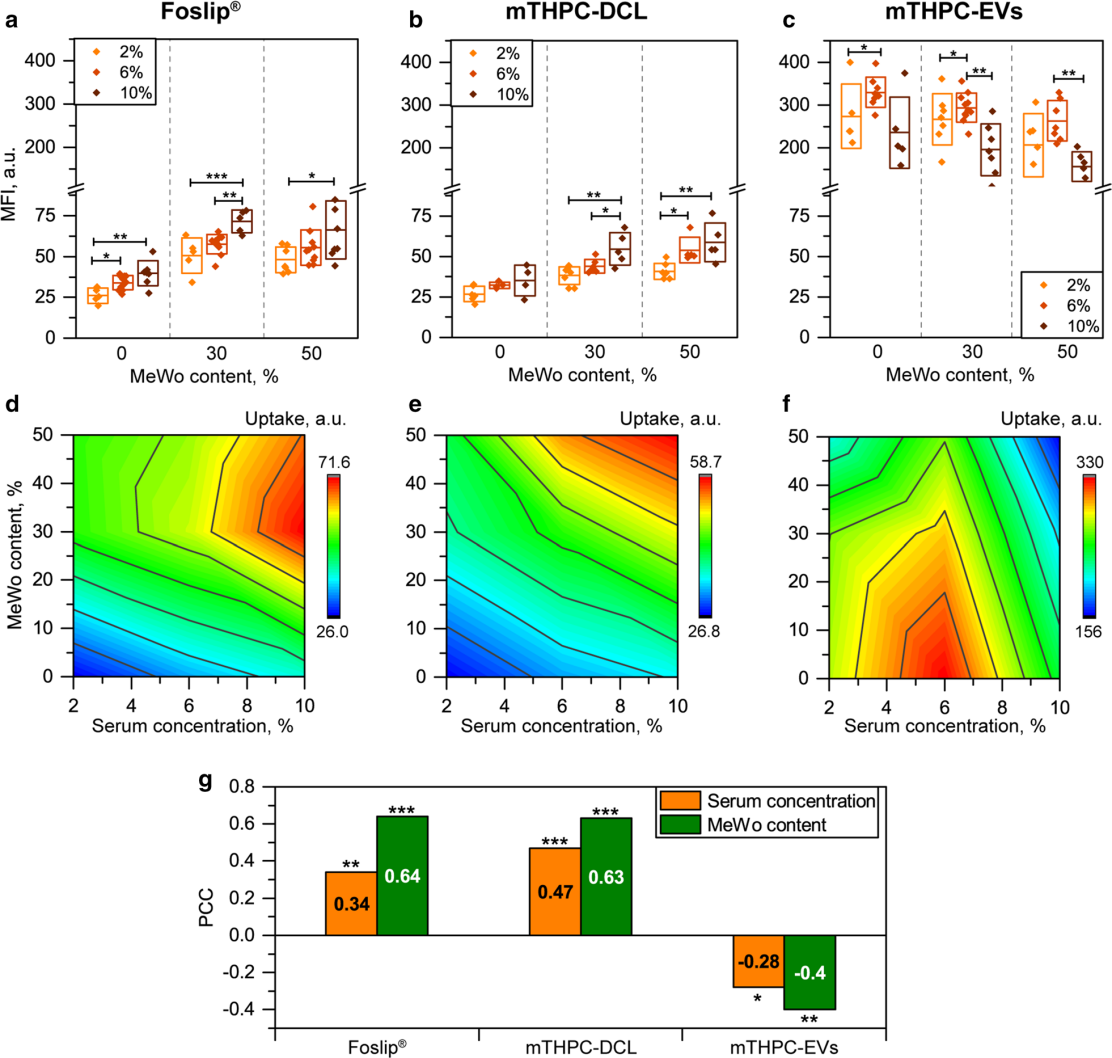
The effect of serum concentration on the cellular uptake of mTHPC-loaded nanovesicles in co-culture spheroids. **a–c** Cumulative mTHPC uptake in spheroids incubated in media supplemented with 2% ( ), 6% ( ) and 10% ( ) of FBS in the function of MeWo content. mTHPC uptake was measured as the mean fluorescence intensity of individual cells after 24 h incubation with NPs. **d–f** 2D contour plot of mTHPC uptake in the function of serum concentration and content of MeWo cells in spheroids. **g** Pearson correlation coefficients (PCC) between mTHPC uptake and serum concentration in culture medium (  ) or MeWo content in spheroid ( ). The concentration of mTHPC was 4.5 µM. * *p* < 0.05; ** *p* < 0.01 and *** *p* < 0.001

It is well-known that the behavior of lipid-based nanoparticles strongly depends on the presence of serum. To study the influence of fetal bovine serum (FBS) concentration on the cellular uptake of nanovesicles in spheroid, we incubated spheroids with mTHPC NPs for 24 h in the presence of 2%, 6%, and 10% FBS (Fig. 10). The increase of FBS concentration resulted in better cellular uptake of both Foslip^®^ and mTHPC-DCL (Panels A&B). Serum proteins were reported to destabilize Foslip^®^ vesicles promoting the rapid release of mTHPC [14, 30]. In turn, mTHPC-DCLs have similar lipid content as Foslip^®^, thus it is anticipated that mTHPC-DCLs will be rapidly destroyed in serum, releasing mTHPC/cyclodextrin nanoshuttles [16]. On the contrary, the effect of FBS on the uptake of mTHPC-EVs was quite ambivalent (Fig. 10c). Remarkably, the highest mTHPC uptake was demonstrated in medium supplemented with 6% FBS, while further increase in FBS concentration resulted in a gradual decrease of mTHPC uptake, in particular for co-culture F5M2 and F5M5 spheroids (*p* < 0.01). Previously, mTHPC-EVs demonstrated unusual behavior upon the interaction with serum, namely shrinking of vesicles without loss of their integrity [14, 18].

Furthermore, we plot 3D contour plots of cumulative cellular uptake as a function of MeWo content and FBS concentration (Fig. 10d–f) to assess the additive effect of both parameters. Finally, to compare the impact of serum concentration and MeWo content for each type of nanovesicles, PCC was calculated for each NP (Fig. 10g). As seen from the contour plot, the highest uptake (red zone) of Foslip^®^ and mTHPC-DCL was observed in the top-right corner of the plot demonstrating a strong positive correlation of MFI with both MeWo content and FBS concentration (Fig. 10d, e, respectively). As anticipated, cellular uptake of natural-derived EVs was less sensitive to FBS concentration (PCC = – 0.28, weak negative correlation, *p* = 0.024) and demonstrated a negative correlation with MeWo content in FaDu spheroids (PCC = – 0.4, moderate negative correlation, *p* = 0.001) (Fig. 10g).

**Fig. 11:**
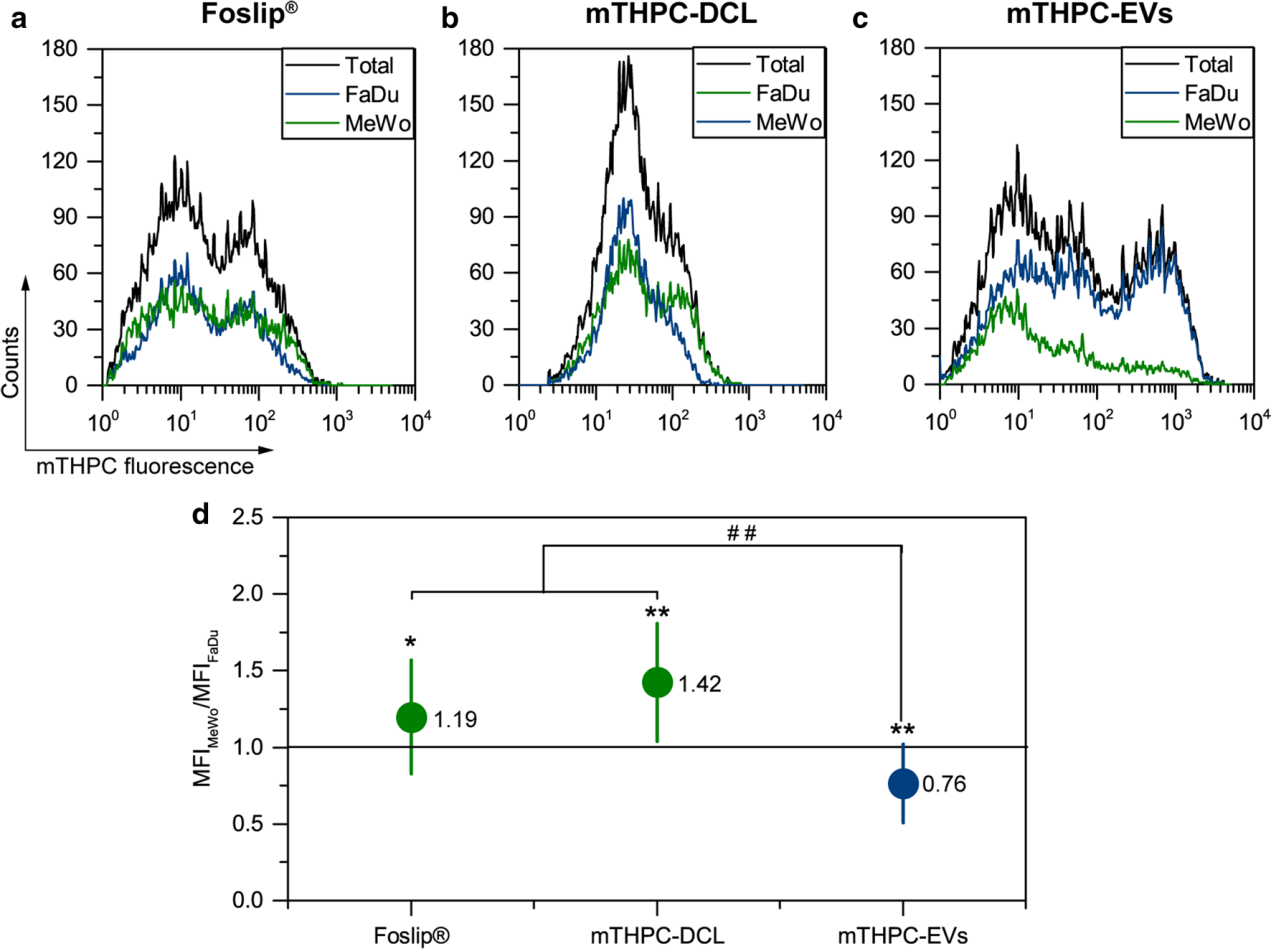
The selectivity of mTHPC NPs against MeWo and FaDu cells. **a–c** Typical flow cytometry histograms of total (black), FaDu (blue) and MeWo (green) populations of cells from dissociated F5M5 spheroids treated with (**a**) Foslip^®^, (**b**) mTHPC-DCLs and (**c**) mTHPC-EVs. mTHPC concentration was 4.5 µM. Serum concentration – 6%. **d** The calculated ratio MFI of MeWo and FaDu cell populations in co-culture spheroids after 24 h of incubation with mTHPC-loaded NPs. Data presented as mean ± SD [n = 12–19; * *p* < 0.05 and *** *p* < 0.001, using one-sample t-test, *µ* = 1; ^##^ *p* < 0.01, using ANOVA]

Finally, we analyzed the distinct distribution of mTHPC nanovesicles in the populations of tumor FaDu cells and MeWo fibroblasts in spheroids (Fig. 11a–c). We also calculated the ratio between the MFI of FaDu and MeWo in co-culture F5M2 and F5M5 spheroids (Fig. 11d). According to the obtained data, Foslip® and mTHPC-DCL were selective to MeWo fibroblasts (the MFI ratio was 1.19 and 1.42, respectively), while mTHPC-EVs, on average, were better accumulated in FaDu tumor cells (the MFI ratio was 0.76). Hence, the nanoformulations, which are selective to MeWo cells, demonstrated in total better uptake in stroma-rich spheroids, confirming the correlations between MFI and MeWo content. As we recently reported, free mTHPC was selective against FaDu cells in co-culture FaDu/MeWo spheroids [8].

### PDT

**Fig. 12:**
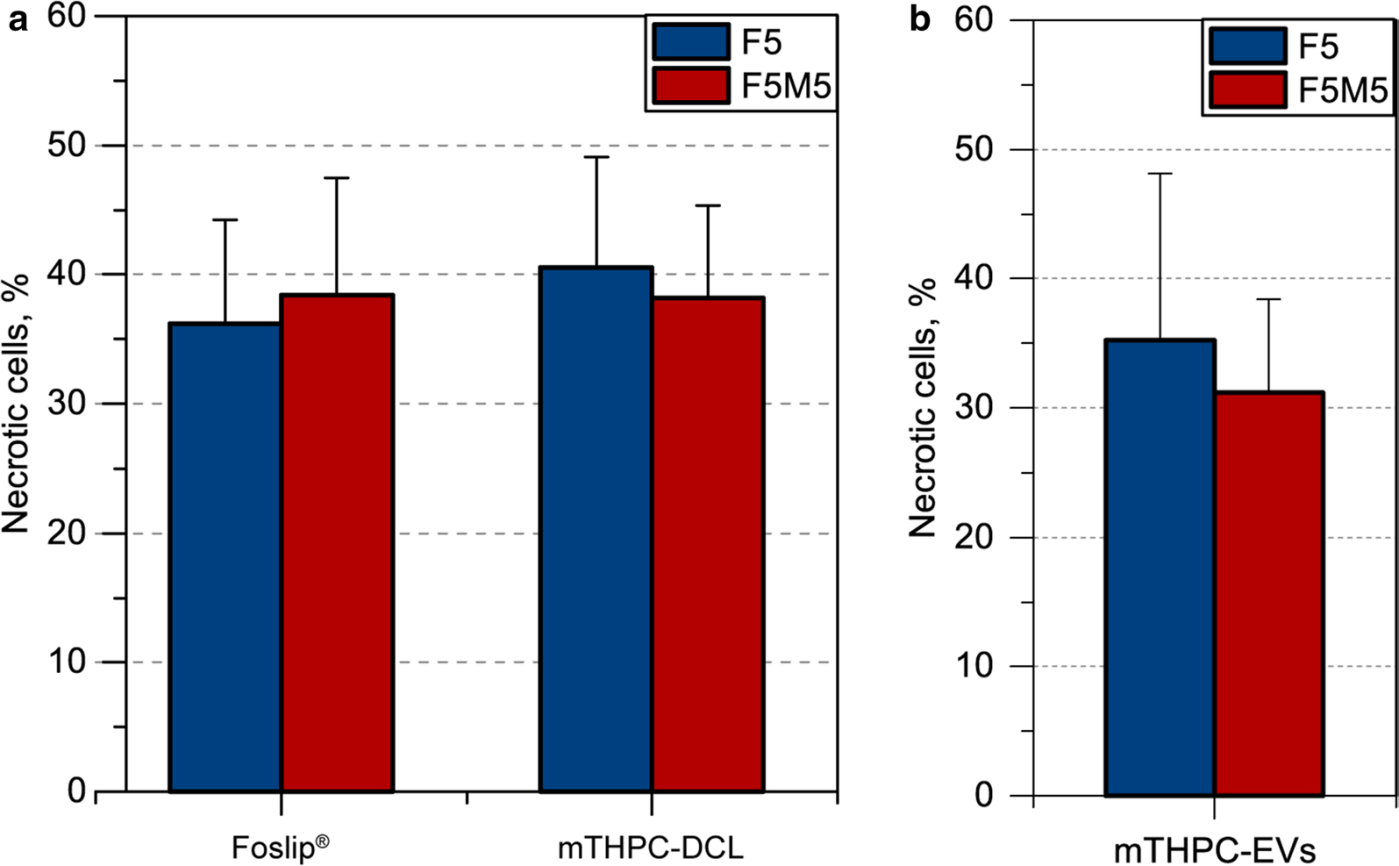
Photodynamic efficiency of mTHPC-loaded NPs in co-culture spheroids. The percentage of necrotic cells 6 h after PDT treatment of (  ) FaDu monoculture and (  ) FaDu:MeWo (F5M5) co-culture spheroids with **(a)** Foslip^®^ and mTHPC-DCL (20 J/cm^2^) and (**b**) mTHPC-EVs (5 J/cm^2^). The concentration of mTHPC was 4.5 µM. Data presented as mean ± SD [n = 3–5; * *p* < 0.05, using two-sample t-test]

Finally, mTHPC-loaded NPs were further tested in terms of photoinduced cell toxicity. Phototoxicity was evaluated by means of flow cytometry in F5 and F5M5 spheroids after 24 h incubation with NPs and 6 h post successive red-light irradiation (Fig. 12). Obviously, mTHPC-EVs possess much higher PDT efficiency than Foslip^®^ and mTHPC-DCL. Indeed, we observed 35.3% ± 12.9% of necrotic cells in stroma-poor F5 spheroids incubated with mTHPC-EVs after irradiation with already 5 J/cm^2^ (Fig. 12b), while it required 20 J/cm^2^ to damage a similar amount of cells in spheroids treated with Foslip^®^ and mTHPC-DCL (36.2% ± 7.9% and 40.5% ± 8.5%, respectively) (Fig. 12a). Of note, toxicity in control (no light, NL) groups did not exceed 15%. Concerning the efficiency of free drug, we recently demonstrated that PDT conducted in monoculture F5 spheroids with 20 J/cm^2^ resulted in only 21.9% ± 4.6% of necrotic cells [8]. Meanwhile, in stroma-rich F5M5 spheroids, we observed a similar PDT effect (*p* > 0.05) for all types of mTHPC-loaded nanovesicles. As we recently demonstrated [8], PDT (20 J/cm^2^) of stroma-rich FaDu/MeWo spheroids exposed to free mTHPC resulted in a higher fraction of dead cells than monoculture F5 spheroids (37.8% ± 5.9% vs. 21.9% ± 4.6, *p* < 0.05). According to this, the PDT efficiency of free mTHPC is similar to that of Foslip^®^ and mTHPC-DCL in stroma-rich F5M5 spheroids. Thus, we can conclude that lipid-based NPs are likely to be less sensitive to the changes of TME, than free drug and could be efficiently used in a wide range of clinical situations.

## Conclusion

In the present work, we studied the behavior of lipid-based mTHPC nanovesicles in 3D stroma-rich spheroid model of HNSCC. Generally, TME is considered as a major challenge for NPs delivery and is one of the main reasons causing the huge gap between preclinical screening and clinical applications. In our study, TME was recapitulated using recently developed co-culture HNSCC spheroids consisting of FaDu tumor cells and MeWo CAFs [8]. In the case of stroma-rich F5M5 spheroids, the presence of 50% CAFs resulted in the visually lower E-cadherin expression and obviously higher expression of vimentin and collagen. The loss of E-cadherin and expression of vimentin in tumor tissue is associated with higher metastatic risk [31], and correlates with poor prognosis for HNSCC patients [32]. Thus, co-culture spheroids represent a valuable model of avascular stroma-rich HNSCC microenvironment.

According to the literature data, most clinical HNSCC tumors (60–70%) are rich in CAFs [33, 34] and they are the primary source of ECM components providing significant physical resistance for effective drug delivery [7, 26]. Using spheroids with increasing stroma content (0%, 30% and 50% of MeWo cells in F5, F5M2 and F5M5 spheroids, respectively), we investigated how stroma affects the accumulation, penetration and photodynamic efficiency of most potent lipid-based mTHPC nanoformulations. Despite the general consideration of stroma as a physical barrier, the penetration of NPs (normalized to spheroids’ size) was constant irrespective of the percentage of stromal components. Moreover, the uptake of nanovesicles based on the conventional liposomes (Foslip® and mTHPC-DCL) was significantly higher in spheroids with a high amount of stroma components. Also, we found that uptake of Foslip® and mTHPC-DCL was higher in serum-rich media, recapitulating conditions of intravenous administration. Nevertheless, PDT-induced photokilling of lipid-based NPs was independent on the amount of stroma in spheroids displaying the relationship between photoinduced efficiency of nanoformulations and NPs’ penetration.

Finally, we performed a head-to-head comparison of lipid-based mTHPC-loaded NPs. Both mTHPC-EVs and mTHPC-DCLs were considered as advanced nanodelivery systems, however head-to-head comparison of them is not currently available. The physical-chemical characteristics of chosen lipid-based NPs (size, charge) were previously reported [14, 15, 18]. Here, we focused on the comparison of their loading capacity and behavior in spheroids. According to our results, naturally-derived EVs are the most efficient lipid-based nanoformulations of mTHPC. Due to the extremely high drug loading, the total uptake of mTHPC-EVs in spheroids is ten times higher than that for other NPs. Despite the preferable accumulation of mTHPC-EVs on the peripherical cell layers, spheroids exposed with mTHPC-EVs required four-times lower light dose to obtain a similar therapeutic effect as mTHPC-DCL and Foslip® at 24 h. Given the uptake kinetics and penetration of nanovesicles in spheroids, we may expect a better PDT outcome of mTHPC-DCLs at short drug-light intervals (DLIs) (3 or 6 h) distributing drugs uniformly across the tumor, while for mTHPC-EVs the optimal DLI should be 15 h or 24 h. Moreover, the limited penetration of Foslip® into the tumor tissues allows us to predict partial PDT response. All these results confirm our recent *in vivo* observations in xenografted HT29 (human colon adenocarcinoma) tumor-bearing animals [16, 18].

Overall, the present work reports an advanced methodology (e.g., advanced image processing, flow cytometry analysis) for the investigation of the behavior of photoactive NPs in 3D multicellular tumor models. The developed co-culture 3D model of HNSCC allows delineation of the role of stromal components on NPs behavior in avascular stroma-rich HNSCC tumors, offering a substantial advantage over in vivo tumor models. The analysis of NPs penetration and uptake in the function of stromal content provides an important evaluation parameter of therapeutic delivery systems and allows better optimizing of NPs design for *in vivo* biodistribution studies. Moreover, the kinetics of NPs penetration in spheroids would enable the prediction of the optimal DLI for the better therapeutic PDT response. In total, the results of the present study enlarge our understanding of how stroma components affect the delivery and photodynamic activity of NPs.

## Materials and methods

### Materials

mTHPC and its liposomal formulation (Foslip^®^) were kindly provided by biolitec research GmbH (Jena, Germany). The stock solution of mTHPC (2 mM) was prepared in ethanol and kept at 4 °C in the dark. Foslip^®^ is based on dipalmitoylphosphatidylcholine (DPPC) and dipalmitoylphosphatidylglycerol (DPPG) and mTHPC with drug:lipid ratio of 1:12 (mol/mol) and DPPC:DPPG ratio 9:1 (w/w). Foslip^®^ powder was reconstituted in water for injection to obtain a 2 mM mTHPC stock solution. The hydrodynamic diameter of liposomes was 114.2 ± 1.0 nm [16].

Heptakis(2,3,6-tri-O-methyl)-β-cyclodextrin (TM-β-CD; product code CY-2003,34; molecular weight 1429.6 Da) was purchased from CYCLOLAB R&D. Ltd., (Budapest, Hungary). DPPC and DPPG were purchased from Sigma (Saint-Quentin Fallavier, France).

### Preparation of mTHPC-DCL

Drug-in-cyclodextrin-in-liposome (mTHPC-DCL) nanoconstructs were prepared by the thin lipid film hydration method, as described previously [15]. Briefly, inclusion complexes of mTHPC with TM-β-CD were formed using the solvent co-evaporation method in ultrapure water. DPPC/DPPG liposomes loaded with mTHPC were prepared by membrane extrusion technique according to the previously published procedure yielding unilamellar liposomes [35]. These liposomes contained DPPC and DPPG at a molar ratio 9:1 with a final lipid concentration of 15 mg/mL. To obtain mTHPC-DCLs, mTHPC was added at the step of the preparation of lipid mixture at a molar drug:lipid ratio 1:15 and mTHPC/TM-β-CD inclusion complexes were encapsulated at the lipid film hydration step. The purification of mTHPC-DCLs from the non-encapsulated mTHPC/TM-β-CDs in the medium was performed using a minicolumn chromatography technique [36]. The hydrodynamic diameter of mTHPC-DCLs was 122.9 ± 1.1 nm (PDI = 0.040 ± 0.013), while the zeta-potential was − 37.5 ± 1.6 mV [16].

### Preparation of mTHPC-EVs

The detailed description of mTHPC-EVs production and isolation was described previously [18]. Briefly, turbulence-triggered EV production and loading were carried out in 1 L bioreactor consisting of human umbilical vascular endothelial cells (HUVEC) by replacing the complete medium by 400 mL of serum-free DMEM medium containing 100 µM of free mTHPC. Spinner flasks of bioreactor were then submitted to rotation at 122 RPM during 4 h [37]. After that, the supernatant was collected and submitted to purification for EV isolation.

EVs were washed and isolated from the conditioned culture medium with a differential (ultra)centrifugation method based on the previously described protocol by Théry et al. [38]. First, cell debris was eliminated by 2000*g* centrifugation for 10 min. The total population of EVs (containing both microvesicles and exosomes) was isolated in a single 100,000 g step for 1 h. mTHPC-EVs were resuspended in serum-free medium and characterized by nanoparticle tracking analysis (NTA 3.2 Software, Malvern Instruments, UK). mTHPC concentration was estimated by LS55 spectrofluorometer (Perkin Elmer, Waltham, MA, USA). The hydrodynamic diameter of mTHPC-EVs was 202.8 ± 12.5 nm [18].

### Cell lines

The FaDu (human pharynx squamous cell carcinoma) cell line was purchased from ATCC (Cat. No: ATCC1 HTB-43™). Cells were cultured in phenol red-free Roswell Park Memorial Institute 1640 medium (RPMI-1640, Invitrogen™, Carlsbad, California, USA), supplemented with 9% (vol/vol) heat-inactivated fetal bovine serum (FBS, Sigma-Aldrich, Saint-Quentin Fallavier, France), penicillin (10,000 IU) streptomycin (10,000 mg/mL) and 1% (vol/vol) 0.2 M glutamin (Invitrogen™, Carlsbad, California, USA). MeWo cells (ATCC HTB-65™), granular fibroblasts, derived from human melanoma [17], were used as CAF. Cells were cultured in Minimal Essential Medium (MEM, Sigma-Aldrich, Saint-Quentin Fallavier, France) supplemented with 9% (vol/vol) of FBS and 1% (vol/vol) 0.1 M sodium pyruvate (Sigma-Aldrich, Saint-Quentin Fallavier, France). Cells were kept as a monolayer culture in a humidified incubator (5% CO_2_) at 37 °C. Cell culture was reseeded every week to ensure exponential growth.

###  Spheroids formation

Spheroids were generated from FaDu cells using the liquid overlay technique, as described previously [8, 39]. Briefly, 100 µL of FaDu cells (5 × 10^4^ cells/ml) and 100 µL of full RPMI medium were added to each well of a 96-well plate previously coated with 1% agarose (w/v in water) and cultured at 37 °C, 5% CO_2_ for 5 days before being taken into experiments. Co-culture spheroids were constructed by seeding FaDu cells (100 µL at 5 × 10^4^ cells/mL) simultaneously with 100 µL of MeWo cells at 2 and 5 × 10^4^ cells/mL (F5M2 and F5M5, respectively). The morphology and size of spheroids were monitored from day 3 after seeding until day 10 by bright field microscopy using an inverted Olympus CK2 microscope (Olympus, Rungis, France). From 8 to 16 spheroids were used for each experimental condition. At day 5 post-seeding, when spheroids reached about 450–550 µm in diameter, they were embedded into resin Shandon™ Cryomatrix™ (ThermoFisher, Waltham, MA, USA), frozen, cut and 10 µm thick sections were further used for fluorescence microscopy and immunohistochemistry analysis.

### Fluorescence staining

To distinguish two types of cells in spheroid co-culture, MeWo cells were pre-stained with a membrane green fluorescent cell marker PKH67 (Sigma-Aldrich, Saint-Quentin Fallavier, France) before seeding with FaDu cells. The pre-staining of MeWo cells was performed following the manufacturer’s instructions. Briefly, the suspension of 10^7^ MeWo cells was washed once with a serum-free medium. The cell pellet was then gently mixed in the dark with 4 µM of PKH67 in the solution provided by the manufacturer for 10 min. The labeling was stopped with the addition of two volumes of fetal bovine serum for 2 min and then washed twice in complete medium before co-seeding with FaDu cells into agarose pre-coated plates. The efficiency and stability of membrane staining were checked by flow cytometry in MeWo cells immediately after staining and in co-cultured spheroids 5 days after seeding.

Before incubation with NPs, spheroids were washed with serum-free RPMI medium. 100 µL of complete medium was carefully removed from the plates and 150 µL of concentrated drug solution, prepared in medium supplemented with 0, 5 or 10% of FBS, was added to spheroids for the final mTHPC concentration of 4.5 µM. The final concentration of FBS in the culture medium was 2, 6 or 10%, respectively. Cells were kept in a humidified incubator (5% CO_2_) in the dark at 37 °C. At appropriate incubation times, from 3 h to 24 h, after washing with serum-free medium, spheroids were embedded into the resin matrix, and 10 µm thick sections were used for fluorescence microscopy. For further analysis, we used the cryosections with the diameter of the spheroid section about 450 µm corresponding to the central part of the spheroid.

### Analytic techniques

#### Spectroscopy

Absorption measurements were recorded with a Lambda 35 spectrometer (Perkin Elmer, USA) and fluorescence measurements were conducted with LS55B spectrofluorometer (PerkinElmer, USA) equipped with polarizers, thermostated cuvette compartments and magnetic stirring for polarization experiments. Fluorescence quantum yield was measured as was previously described (λ_ex_: 416 nm; λ_em_: 652 nm) [40].

#### Histology and immunofluorescence analysis

The frozen sections were fixed with buffered 4% formaldehyde supplemented with sucrose 2% (m/v) for 10 min and rinsed with phosphate-buffered saline (PBS) before staining for immunofluorescence/ immunohistochemical characterization. The extracellular matrix was evidenced by different markers, such as vimentin, fibronectin, and collagen. Epithelial–mesenchymal transition phase was characterized by epithelial marker as E-cadherin. Antibodies for fibronectin, collagen and E-cadherin were provided by ThermoFisher (Waltham, MA, USA) and vimentin antibody by Dako.

For immunofluorescence imaging, fixed cryosections were permeabilized with 0.2% Triton X-100 for 5 minutes, and blocked with a solution of PBS-bovine serum albumin (3% w/v) for 1 h at room temperature, before overnight incubation with primary antibody in blocking solution in a humidified chamber. E-cadherin antibody was diluted at 1:100. Samples were washed extensively before indirect immunostaining with secondary anti-rabbit Alexa 555 conjugated antibody in PBS for 1 h at room temperature. After several washings, samples were mounted with a nuclear counterstaining solution with DAPI (Vectashield with DAPI, Vector laboratories, Burlingame, CA, USA) and then observed by fluorescence microscopy.

Vimentin expression was evidenced by immunohistochemical staining in Benchmark Ultra Automat (Ventana, Tucson, AZ, USA) as previously described in [8]. Vimentin antibody was diluted at 1:200 (Clone V9; Dako, Santa Clara, CA, USA).

#### Flow cytometry

Flow cytometry was used to study the kinetics of mTHPC accumulation in spheroids and the dependence of mTHPC cellular uptake on the concentration of serum in the culture medium. With this purpose, spheroids were incubated with NPs for 24 h in the medium supplemented with 2, 6 or 10% of FBS. In order to dissociate spheroids, they were transferred into a 12-well plate, washed twice with PBS, incubated with twice diluted trypsin-EDTA in PBS (Sigma-Aldrich, Saint-Quentin Fallavier, France). Afterward, the plate with spheroids was protected from light, placed on the rotatory shaker (60 rpm) for 20–25 min and then 3 mL of the complete culture medium was added to inhibit trypsinization. Finally, spheroids were resuspended, centrifuged (1500 rpm, 5 min) and the pellet was resuspended in the fresh serum-free culture medium.

Flow cytometry analysis was performed using FACSCalibur (BD, Franklin Lakes, NJ, USA), equipped with lasers emitting at 488 nm and 633 nm. The fluorescence of PKH67 was detected in the fluorescence channel FL1 with a 533 ± 30 nm filter under the excitation at 488 nm, while the detection of mTHPC fluorescence was performed in FL4 channel with 661 ± 16 nm filter under the excitation at 633 nm. Propidium iodide fluorescence was detected in FL2 with a 585 ± 30 nm filter (excitation at 488 nm). Data analysis was carried out using Flowing Software (Turku Centre for Biotechnology, Turku, Finland).

#### Fluorescence microscopy

Fluorescence images were collected from spheroids cryosections. Fluorescence was observed under an upright epifluorescence microscope (AX-70 Provis, Olympus, Paris, France). PKH67 fluorescence was observed using 460–490 nm excitation bandpass filter associated with a 505 nm dichroic mirror and 510–550 nm emission bandpass filter. The fluorescence images of mTHPC were obtained using the filter set at 405–445 nm excitation associated with a 570 nm dichroic mirror and a 590 nm long-pass emission filter for fluorescence measurements.

The analysis of images was performed with ImageJ (NIH, USA) software. To estimate the penetration profiles of mTHPC-loaded NPs in spheroids, custom macros for ImageJ was used [24]. Briefly, the spheroid area was divided into 100 concentric rims with a linearly decreasing diameter. After that, the mean intensity of pixels in each rim was calculated. The final profiles were plotted as mean ± standard deviation (SD) from different cryosections (n = 4–9). The estimated penetration profiles were normalized to the mean radius of spheroids. The cumulative accumulation curves were obtained by the integration of penetration profiles. The final cumulative curves were normalized and plotted as a mean tendency. The mid penetration depth (d_1/2_) was calculated from cumulative accumulation curves as the depth of 50% of mTHPC uptake. The quantitative analysis of penetration profiles was performed in Origin software (OriginLab, Northampton, MA, USA).

### Statistics

The data from at least three independent experiments are presented as mean ± SD. One-tailed t-test was used for statistical analysis of mTHPC selectivity (ratio of mean fluorescence intensities) with µ = 1 as H_0_. An unpaired, two-tailed t-test was used for statistical analysis of two groups. Analysis of Variance (ANOVA) followed by Tukey’s multiple comparisons test was used for comparison of three or more groups. Data analysis, including the estimation of Pearson correlation coefficients, was carried out with the Origin software.

## Data Availability

The datasets used and/or analyzed during the current study are available from the corresponding author on reasonable request.

## Abbreviations

NP: Nanoparticle
TME: Tumor microenvironment
ECM: Extracellular matrix
HNSCC: Head and neck squamous cell carcinoma
CAF: Cancer-associated fibroblast
mTHPC: 5,10,15,20-Tetrakis(3-hydroxyphenyl)chlorin, temoporfin
PS: Photosensitizer
PDT: Photodynamic therapy
mTHPC-DCLs: mTHPC-loaded drug-in-cyclodextrin-in-liposomes
mTHPC-EVs: mTHPC-loaded extracellular vesicles
F5: Monoculture FaDu spheroids (5000 cells per well)
F5M2: Co-culture FaDu/MeWo spheroids (5000 FaDu and 2000 MeWo cells per well)
F5M5: Co-culture FaDu/MeWo spheroids (5000 FaDu and 5000 MeWo cells per well)
PCC: Pearson correlation coefficient
DPPC: Dipalmitoylphosphatidylcholine
DPPG: Dipalmitoylphosphatidylglycerol
d_1/2_: Mid penetration depth
MFI: Mean fluorescence intensity
CV: Coefficient of variation
FBS: Fetal bovine serum
TM-β-CD: Heptakis(2,3,6-tri-O-methyl)-β-cyclodextrin
RPMI-1640: Roswell Park Memorial Institute 1640 medium
MEM: Minimal Essential Medium
PBS: Phosphate-buffered saline
SD: Standard deviation
ANOVA: Analysis of variance
